# The Effect of Spinosad on the Oak Lace Bug *Corythucha arcuata* (Hemiptera: Tingidae)—A Preliminary Study Performed Under Laboratory Conditions

**DOI:** 10.3390/insects15100815

**Published:** 2024-10-16

**Authors:** Ciprian George Fora, Artúr Botond Csorba, Adalbert Balog

**Affiliations:** 1 Faculty of Engineering and Applied Technologies, University of Life Sciences “King Michael I” from Timișoara, Calea Aradului 119, 300645 Timișoara, Romania; 2 Department of Horticulture, Faculty of Technical and Human Sciences, Sapientia Hungarian University of Transylvania, Aleea Sighișoarei 2, Târgu Mureș, 547367 Corunca, Romania; csorba.artur@ms.sapientia.ro

**Keywords:** spinosad, lambda-cyhalothrin, mortality, effectiveness, oak forests, pest control, Europe

## Abstract

**Simple Summary:**

The oak lace bug is a rapidly spreading oak forest pest in Europe. These days, chemical control has been largely reduced in favor of biological methods due to the high biodiversity values of these forests. Here, we tested for the first time the effect of biological compound spinosad on lace bug mortality and found that this can be an effective method of controlling this insect pest.

**Abstract:**

The effect of biopesticide compound spinosad in different concentrations was tested for the first time under laboratory conditions against the rapidly spreading forest pest, oak lace bug (*Corythucha arcuata*, Say 1832), and its effects were compared with the synthetic pesticide lambda-cyhalothrin. These results revealed a significant effect of spinosad at 2 mL/4 L and 2 mL/2 L water concentrations against *C. arcuata* nymphs. The mortality rate after 3 days was similar to synthetic insecticide effects and reached 94% and 98%, respectively. Overall, it can be concluded that spinosad is an effective biological method to control oak lace bug; treatments under field conditions should consider the high diversity of other insects in oak forests.

## 1. Introduction

The oak lace bug (*Corythucha arcuata*, Say 1832) has been reported in Europe since 2000 as a new invasive sap-sucking insect, causing sever infestation in European oak forests [1]. Its first report in Central Europe in 2013 was followed by new reports and infestations in southeastern Europe [2]. Nowadays, its frequency in Romanian oak forests is high since its first recording in 2016, and it is increasing from year to year [3]. In the western Romanian Carpathians, in 2019, adults of *Corythucha arcuata* were observed in the herbaceous blanket of Bihor Peak at 1849 m a.s.l., and in the southern Romanian Carpathians, in 2024, in the herbaceous blanket of Baba Mare Peak at 2292 m a.s.l. (unpublished observations, Fora). Therefore, it is clear that the ecological plasticity of the species is high. Nymphs, adults of the lace bug, cause small, later expanding, and merging spots by sucking on the underside of leaves on the upper leaf surface. These spots often cover the whole leaf surface during the summer period [2]. Due to the severe drought occurring in European oak forests in recent years, such insect damages can cause a high loss in forests stands, making this problem even more complex and acute [4]. The possible control of this invasive insect included both chemical and biological methods [3,4]. The use of synthetic pesticides (e.g., contact insecticide (Alfametrin 10CE) and the systemic insecticide (APIS 200 SE)) revealed a reduction in the nymph population that varied from 91% to 96%; however, it was also reported that the residual population was sufficient to cause re-infestations over time. These new results also showed that the reinfestation occurred even more quickly after contact insecticide sprays (22 days after treatment) and slowly after systemic insecticide sprays (more than a month after treatment) [3,4]. As chemical insecticides are less and less accepted in forest protection, alternative active ingredients, preferably environmentally friendly and at the same time more effective, have to be found in the coming short period to protect the forest. Using biological control, a high rate of mortality among overwintering individuals (65%) has been found, with 19% demonstrating involvement in infections by various entomopathogenic fungi with 70% belonging to *Beauveris pseudobassiana* [5,6]. Several other studies have also demonstrated positive correlations between the population expansion of this insect in Europe and climatic changes (i.e., warming climate) [1,6,7] whilst at the same time also revealing potential natural enemies [8,9]. Still, its control using biological insecticides remains insufficiently assessed. Therefore, during the present study, we aimed to test the effect of spinosad (Laser™ 240 SC), a new chemically synthesized insecticide allowed for use in forest crops, against the oak lace bug and compare the effect with the synthetic compound lambda–cyhalothrin (Karate Zeon 5 CS). Because these tests were made for the first time with this compound against this pest, only laboratory studies were allowed and made, and the preliminary results are presented here.

## 2. Materials and Methods

**Figure 1:**
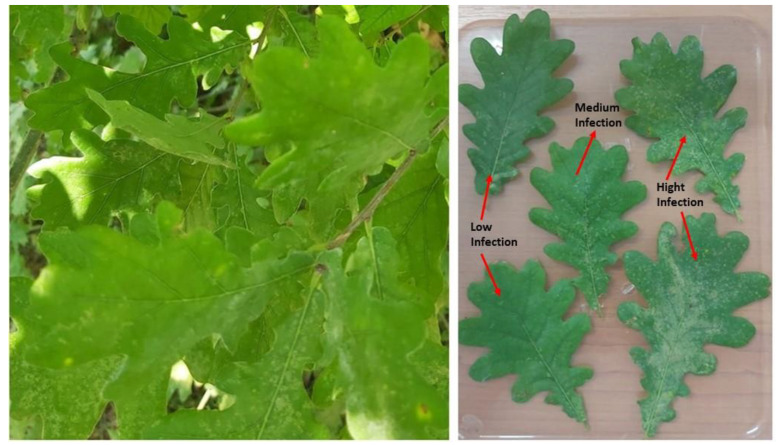
Different oak leaves infestation (low, medium and high) by the oak lace bug.

In the 2024 growing season, oak forests in the Banat region, Timiș County, Romania were visited, and the infestation rate of *C. arcuata* was assessed. Because all oak forests were severely infested, leaf samples colonized with *C. arcuata* were collected. Randomly selected oak trees (*Quercus robur*) were searched, and from each tree, five leaf samples populated with *C. arcuata* nymphs were collected and returned to the laboratory for testing (Figure 1). Assessing the efficacy of the biological insecticide spinosad 240 g/L, lab experiments were performed involving treatments with spinosad solution at different concentrations; a control using distillated water and a positive control using lambda-cyhalothrin (recommended against this pest by authorities in USA) were used (Table 1).

Spinosad (Laser™ 240 SC) has a broad effectiveness on chemically synthesized insecticides, and it is allowed in biological crops; it acts on the nervous system of insects, causing their paralysis [10]. The active substance spinosad does not generate forms of resistance; it was approved in Romania against Thrips (*Frakliniella occidentalis*, *Thrips tabaci*), Mine fly (*Liriomyza trifolii*), Cherry fly (*Rhagoletis cerasi*), Plum worm (*Cydia funebrana*), Stone wasp (*Eurytoma schreinerii*), Colorado beetles (*Leptinotarsa decemlineata*), Marbled miner (*Phyllonorycter blancardella*, *P. corylifoliella*), Apple worm (*Cydia pomonella*), Grape vine moth (*Lobesia botrana*) and Cabbage moth (*Plutella maculipenis*). Its effect against *C. arcuata* is here tested for the first time [11]. 

In total, five variants and four replications from each variant and a total of 20 leaves/replications/variants were used. Before treatment, the existing nymphs were counted on each individual leaf sample, and spraying was immediately performed. The treated leaf samples were placed in transparent plastic boxes with perforated lids and then kept for observations in the laboratory with natural light and a temperature of 22 °C. Efficacy was calculated by the Henderson–Tilton formula [12] at 1 and 3 days after treatment, respectively. 

### Data Analyses

Altogether, 3959 nymphs of *C. arcuata* were counted and used in the experiment in five variants with four replications each. The distribution of the data was examined by the Kolmogorov–Smirnov test. The original data were not distributed normally; therefore, the nonparametric Kruskal–Wallis text was used followed by Mann–Whitney U tests, which were used to compare the treatments. Means with a different letter on the figures represent statistically significant differences.

## 3. Results

**Figure 2:**
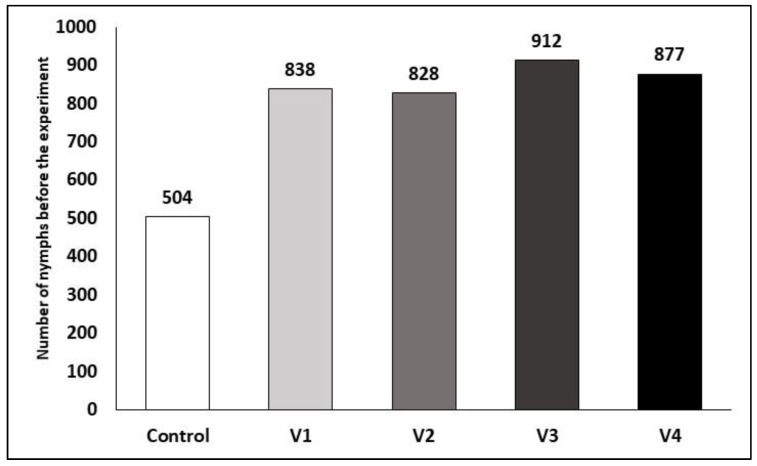
Mean number of *C. arcuata* nymphs before the treatments.

The mean number of each variant is shown in Figure 2. After the treatments, the corrected efficacy was calculated using the Henderson–Tilton formula.

### 3.1. Efficacy of the Treatments After One Day

**Figure 3:**
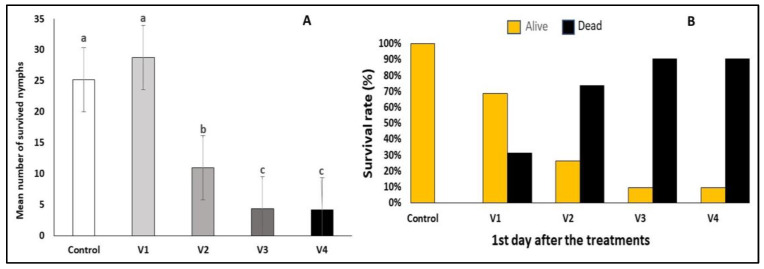
Efficacy of different treatments on *C. arcuata* nymphs after one day (Mann–Whitney test, *p* < 0.05) (**A**). Different letters represent statistically significant differences. Survival rate and corrected efficacy on *C. arcuata* nymphs after one day (Henderson–Tilton’s formula) (**B**).

The application of the pyrethroid insecticide lambda–cyhalothrin (Karate Zeon 50 CS, V4) resulted in a 90% mortality rate of *C. arcuata* nymphs within 24 h post-treatment. Similarly, treatments with the biological insecticide spinosad demonstrated significant effectiveness in controlling *C. arcuata* nymphs across various concentrations except for one variant (V1-2 mL/6 L water). Statistical analyses revealed no significant differences in mortality rates between Variant 3 (V3) spinosad 2 mL/4 L water and Variant 4 (V4) spinosad 2 mL/2 L water and lambda–cyhalothryn (F = 0.09, *p* ≤ 0.9). At the same time, a low survival rate was detected in treatments V2, V3 and V4 (Figure 3A,B).

### 3.2. Efficacy of the Treatments After Three Days

**Figure 4:**
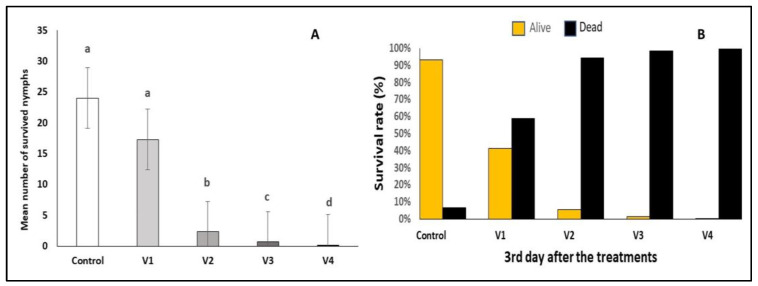
Efficacy of different treatments on *C. arcuata* nymphs after three day (Mann–Whitney test, *p* < 0.05) (**A**). Different letters represent statistically significant differences. Survival rate and corrected efficacy on *C. arcuata* nymphs after three days (Henderson–Tilton’s formula) (**B**).

After three days of exposure to the lambda-cyhalothrin treatment (Karate Zeon 50 CS), the survival rate of *C. arcuata* nymphs was reduced to nearly 0%. In addition, the spinosad treatments demonstrated remarkable efficacy with mortality rates exceeding 94% in Variant 2 (V2—2 mL/4 L water) and 98% in Variant 3 (V3—2 mL/2 L water). Statistical analysis revealed significant differences in mortality rates between the untreated control group and the V2, V3, and V4 variants (Control–V2, F = 16.5, *p* ≤ 0.001; Control–V3, F = 17.7, *p* ≤ 0.001; Control–V4, F = 18.1, *p* ≤ 0.001; V1–V2, F = 11.6, *p* ≤ 0.001; V1–V3, F = 12.9, *p* ≤ 0.001; V1–V4, F = 13.3, *p* ≤ 0.001). Again, a low survival rate after treatments V2, V3 and V4 was observed (Figure 4A,B).

## 4. Discussion

These results, performed under laboratory conditions, revealed the effectiveness of spinosad as a biological insecticide in controlling *C. arcuata* nymphs. The high mortality of 94% and 98% after spinosad treatment using 2 mL/2 L water (V3 in our experiment) would probably be lower, and it is still effective under open field conditions; however, the possibilities of recolonizations of survived nymphs—also reported by previous studies—must be considered when involving field applications [3]. According to these previous studies, the effectiveness of compounds (both biological and synthetic) under field conditions must approach 100%; therefore, additional tests using combinations of different compounds have to be considered. Another similar study tested the insecticidal activity of deltamethrin and imidacloprid, thujone and essential oil of rosemary against the sycamore lace bug (*Corythucha ciliata*). The study also followed mortality rate analyses after one and three days, and it revealed that the most efficient treatment was deltamethrin, which caused almost 100% mortality of both larvae and adults. This was followed by imidacloprid, which caused an 89.6% larval mortality at the recommended concentration, and essential oil of rosemary, which caused 81.7% adult mortality at 1% concentration. The study also revealed that the lowest mortality was observed one day after treatment (41.7%), while the highest was observed three days after treatment (71.3%) [13].

Because oak forests are considered natural reserves and most of the stands are protected in Europe, the significance of biological pest control can be seen as the only method allowed for controlling this dangerous pest. Also, oak forests compared to other tree species harbor one of the highest levels of biodiversity of herbivorous insect species and support the highest associated species richness [11,12,13], the control of such invasive insect including biological methods must be a priority. Other studies revealed that control tactics including cultural methods and combinations of biological methods can make this pest insignificant [2]; therefore, applications of biological insecticides like spinosad under field conditions in the large forests have to be considered. Nematodes tests were also followed to assess the effect on *C. ciliate* and revealed that *Heterorhabditis indica* (HOM1) produced the highest level of infective juveniles in *C. ciliate* [14,15,16,17]. Other similar studies revealed that *Heterorhabditis bacteriophora* did not infect *C. ciliata* nymphs, while *Steinernema carpocapsae* exhibited higher virulence [18]. Such experiments on *C. arcuate* are missing; therefore, additional studies needs to be made to test other biological methods that can be applied in European oak forests.

## 5. Conclusions

Control of the oak lace bug in European oak forests must be prioritized due to its present expansions and concomitant damage. As only biopesticides and predators (including nematodes and parasitoids) are allowed to be used in insect pest control in Europe, an alternative and effective compound spinosad can be usefully employed, which must be applied with full regard to the high biodiversity values of these forests. Combinations of several biological compounds, also reported by other authors, must be applied under field conditions to reach 100% efficacy and thus recolonizations due to any surviving nymphs.

## Figures and Tables

**Table 1:** Different treatments used in the experiment.

Variants	Commercial Name	Active Ingredient	Dosage
Control	-	-	-
V1	Laser 240 SC	spinosad 240 g/L	2 mL/6 L water
V2	Laser 240 SC	spinosad 240 g/L	2 mL/4 L water
V3	Laser 240 SC	spinosad 240 g/L	2 mL/2 L water
V4	Karate Zeon 5 CS	lambda-cyhalothrin 50 g/L	2 mL/10 L water

## Data Availability

All data generated or analyzed during this study were collected by the authors of this publication. The data are available at Figshared: 10.6084/m9.figshare.27232068 (accessed on 1 September 2024).
